# Combined Estrogen Alpha and Beta Receptor Expression Has a Prognostic Significance for Colorectal Cancer Patients

**DOI:** 10.3389/fmed.2022.739620

**Published:** 2022-03-14

**Authors:** Geriolda Topi, Souvik Ghatak, Shakti Ranjan Satapathy, Roy Ehrnström, Marie-Louise Lydrup, Anita Sjölander

**Affiliations:** ^1^Division of Cell Pathology, Skåne University Hospital, Lund University, Malmö, Sweden; ^2^Division of Pathology, Department of Translational Medicine, Skåne University Hospital, Lund University, Malmö, Sweden; ^3^Division of Surgery, Department of Clinical Sciences, Skåne University Hospital, Lund University, Malmö, Sweden

**Keywords:** estrogen receptor beta, estrogen receptor alpha, colorectal cancer, CRC disease-free survival, CRC overall survival

## Abstract

We reported that high estrogen receptor beta (ERβ) expression is independently associated with better prognosis in female colorectal cancer (CRC) patients. However, estrogen receptor alpha (ERα) is expressed at very low levels in normal colon mucosa, and its prognostic role in CRC has not been explored. Herein, we investigated the combined role of ERα and ERβ expression in the prognosis of female patients with CRC, which, to the best of our knowledge, is the first study to investigate this topic. A total number of 306 primary CRCs were immunostained for ERα and ERβ expression. A Cox regression model was used to evaluate overall survival (OS) and disease-free survival (DFS). The combined expression of high ERβ + negative ERα correlates with longer OS (HR = 0.23; 95% CI: 0.11–0.45, *P* <0.0001) and DFS (HR = 0.10; 95% CI: 0.03–0.26, *P* < 0.0001) and a more favorable tumor outcome, as well as significantly higher expression of antitumorigenic proteins than combined expression of low ERβ + positive ERα. Importantly, we found that low ERβ expression was associated with local recurrence of CRC, whereas ERα expression was correlated with liver metastasis. Overall, our results show that the combined high ERβ + negative ERα expression correlated with a better prognosis for CRC patients. Our results suggest that the combined expression of ERα and ERβ could be used as a predictive combination marker for CRC patients, especially for predicting DFS.

## Introduction

The physiological effects of estrogens are mediated by two main receptors, estrogen receptor alpha (ERα) and estrogen receptor beta (ERβ), which belong to the nuclear receptor family and are encoded by two different genes, *ESR1* (ERα) and *ESR2* (ERβ) (1, 2). These receptors are implicated in different types of cancer, including colorectal cancer (CRC) (1–3).

ERβ is the predominant ER in normal colon mucosa, and its expression is reduced during tumor progression (4). Previous research has reported association of ERβ expression with CRC survival (5, 6). We recently reported that high nuclear ERβ expression is independently associated with better prognosis in female CRC patients and associated with hormonal status but not with lifestyle indicators (7). Furthermore, we investigated the antitumor effects of ERβ induction in colon cancer cells and in an *in vivo* zebrafish xenograft model (8). On the other hand, ERα is expressed at very low levels in normal colon mucosa (1, 2), and few studies have reported its prognostic role in CRC survival (9–11). Evidence shows that the manipulation of estrogen signaling to inhibit ERα and stimulate ERβ may have preventive and therapeutic effects for obesity-associated colon cancer (12, 13). However, the relationships among estrogen hormones, reproductive factors, and CRC remain unclear and await further investigation (14).

Many mutations and proteins have been implicated in CRC progression. *KRAS* mutation status is reported to be an important prognostic and treatment marker in CRC, and screening for *KRAS* mutations is now mandatory for metastatic colon cancer before treatment with therapies that target the EGFR pathway (15–17). Furthermore, the activation of the Wnt/β-catenin pathway plays a crucial role in CRC development and progression (18). In addition, high cyclooxygenase-2 (COX-2) expression in CRC correlates with poor prognosis *via* the effect of prostaglandin E_2_ (PGE_2_) (19). 15-Hydroxyprostaglandin dehydrogenase (15-PGDH) is the key enzyme in PGE_2_ catabolism and is often downregulated in CRC, while its upregulation has been shown to lead to a better prognosis in CRC (20–22). The G protein-coupled receptors cysteinyl leukotriene receptors 1 and 2 (CysLT_1_R and CysLT_2_R, the receptor for LTD_4_ respectively LTC_4_) are implicated in the prognosis of CRC (23). Patients with low CysLT_1_R and high CysLT_2_R expression levels have better survival than those with high CysLT_1_R and low CysLT_2_R expression levels (23).

In this study, we aimed to investigate the prognostic significance of the combined expression of ERα and ERβ in female CRC patients and to explore their correlations with other tumor promoter or suppressor proteins and hormonal status.

## Materials and Methods

### Study Populations

The study included a cohort of female patients who were diagnosed with CRC and operated between January 1, 2008, and June 30, 2012. This investigation included 269 patients with available data on clinical information, tumor characteristics, hormonal status as well as ER, ER, KRAS, CysLT_1_R, CysLT_2_R, COX-2, 15-PGDH, β-catenin, Mucin-2 and PGD2 synthase expression in CRC tissue. The study population is briefly described in the Supplementary Materials. Details about the study design, patient follow-up and data collection are provided elsewhere (7).

### Immunohistochemistry (IHC)

Tumor samples were retrieved and incorporated into tissue microarray (TMA) blocks based on the protocol described earlier (7). The tissues were stained with specific antibodies for the expression of ERα ERβ and other proteins of interest (Supplementary Material). Two independent investigators (GT and RE), blinded to the patient and tumor characteristics, evaluated the staining immunoreactivity using the immunoreactive score (IRS) with a range 0–9, which was calculated as a multiplication of staining intensity (0 = negative, 1 = weak, 2 = moderate and 3 = strong) with percentage of positive stained cells (1 = <10%, 2 = 11–50% and 3 = >50%) (7). The staining intensity was determined based on the criteria of Konstantinopoulos et al. (4), which are described in the Supplementary Materials. For ERα and ERβ expression, only the nuclear staining intensity was taken into consideration, based on which they were also scored as categorical variables, respectively low/high and negative/positive expression (**Figure 2A**). Briefly, negative and weak ERβ staining were grouped as low expression and moderate and strong ERβ staining as high expression (7). Because ERα is very little expressed in the normal colonic mucosa (1, 2), we defined its expression as positive if more than 10% of the nuclei were stained, regardless the staining intensity. All the other tumor samples that had <10% of the nuclei stained, regardless the staining intensity, were considered to have negative ERα expression. Each tumor sample was in duplicate. Cores with loss of tissue or with only stromal tissue were excluded from the analysis.

### Acquisition of Gene Expression and Clinical Data From the Cancer Genome Atlas (TCGA) Dataset

Normalized RNA sequencing data in transcripts per million (TPM), reverse phase protein array (RPPA) data, and the associated clinical information of the colon adenocarcinoma (COAD) samples were downloaded from the TCGA dataset (https://portal.gdc.cancer.gov/; https://tcpaportal.org/tcpa/; ≤ June 20, 2020). Out of 361 patients, 12 patients missing pathological information, 16 patients with a follow-up period of ≤30 days, and 52 patients with metastasis (stage IV) were eliminated. Thus, 282 patients with clinical information were included in the study. Normalized gene expression and protein expression data from the TCGA-COAD dataset were log2-transformed for further analysis.

### Identification of Independent Prognostic Parameters of Colon Cancer

To identify independent prognostic parameters and to validate the independent prognostic value of ERα and ERβ, univariate and multivariate Cox regression analyses were performed in the TCGA-COAD dataset on the ERα and ERβ gene and protein signature and clinicopathological parameters. Parameters with *P* < 0.05 in the univariate analysis were further included in the multivariate Cox regression analysis. The TCGA samples were divided into high- and low-risk groups according to the optimal cutoffs determined by the Youden Index association criteria and analyzed using Circos visualization package (24).

### Statistical Analysis

The variables were compared between the group of interest using Pearson's χ^2^ test or Fisher's exact test for categorical variables and the Mann-Whitney *U* test or *t*-test for continuous variables. Survival curves, generated *via* the Kaplan-Meier method, were compared between the groups using the log-rank test. Univariate and multivariate Cox proportional hazards regression models were applied, and hazard ratios (HRs) together with 95% confidence intervals (CIs) were calculated to determine the risk of death or cancer recurrence. Receiver operating characteristic (ROC) curves were used to calculate the area under the curve (AUC) to determine the predictive ability of the final model with combined ERβ + ERα expression compared to models with only one ER expression or the basic model. Binary logistic regression model was used to determine the odds ratios (ORs) of having a metastatic event for each unit increase in ERα and ERβ intensity. The estimates with their corresponding 95% CIs were used to build forest plots by the ggplot2 package in R. Statistical analyses were performed using SPSS version 23.0 (SPSS, IBM, Armonk, NY, USA) and GraphPad Prism version 8.0a (GraphPad Software, Inc., San Diego, CA, USA). A two-sided *P* < 0.05 was considered statistically significant.

## Results

### Evaluation of ERα and ERβ Expression in Female CRC Patients

**Figure 1:**
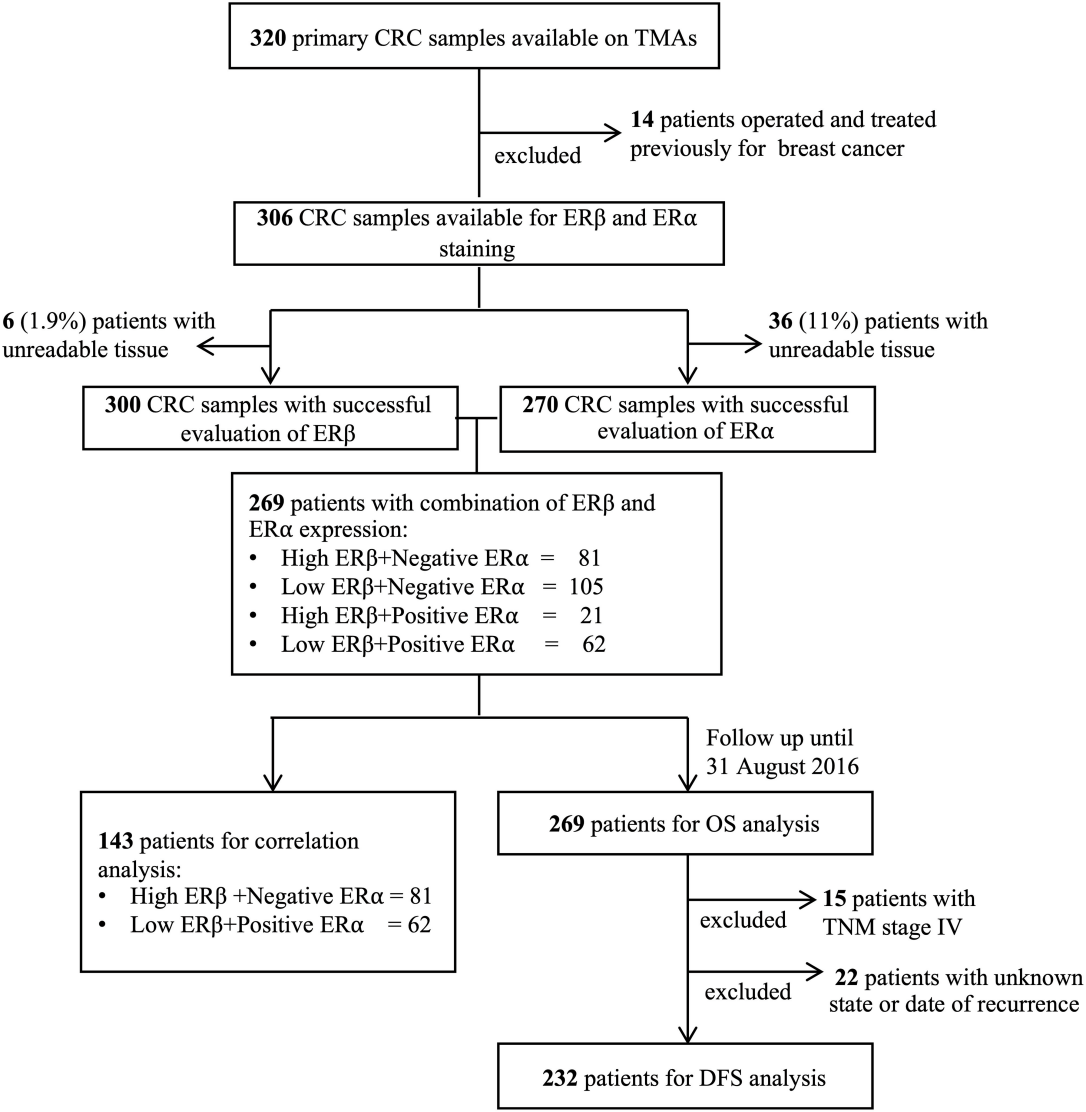
Consort diagram of colorectal cancer patients involved in the study.

**Figure 2:**
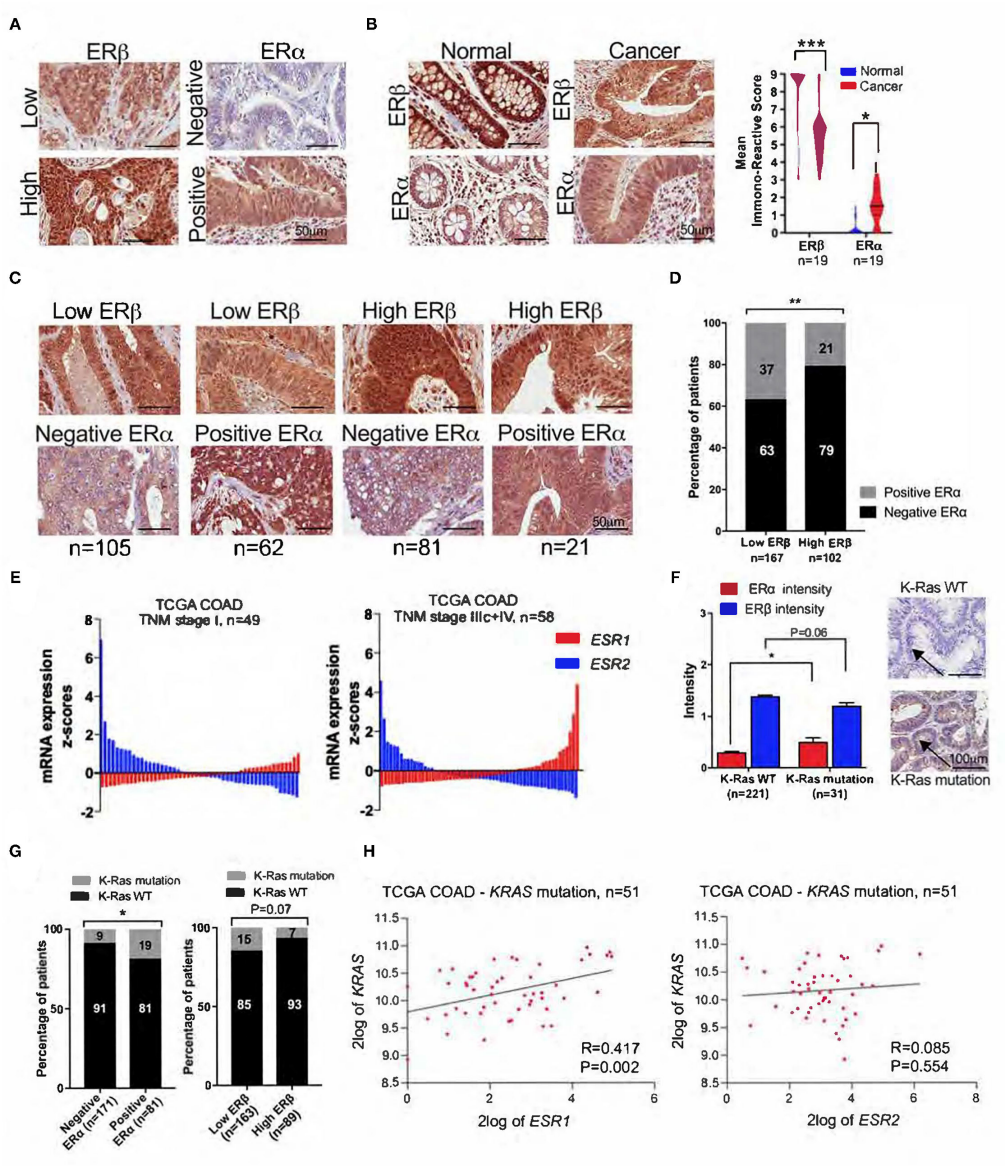
Expression levels of ERα and ERβ in CRC tissue. **(A)** Representative IHC images showing the nuclear expression of ERα and ERβ in CRC tissue. **(B)** Representative IHC images of ERα and ERβ expression in normal and matched cancer tissues, and violin plots showing the distribution of IRSs for ERα and ERβ expression in normal and matched cancer tissues. **(C)** IHC images of CRC tissue in four subgroups of patients with combined ERα and ERβ expression levels. **(D)** The percentage of CRC patients with negative and positive ERα expression according to low and high ERβ expression. **(E)** Waterfall plots of the mRNA expression levels of ESR1 (ERα) and ESR2 (ERβ) in the subgroups of CRC patients with TNM stage I (*n* = 49) and TNM stage IIIc + IV (*n* = 58) from the TCGA-COAD public database. **(F)** Intensity of ERα and ERβ expression in patients with wild-type (WT) and KRAS mutations, together with representative IHC images for KRAS status. The arrows indicate negative and positive staining. **(G)** The percentage of CRC patients with KRAS mutations and KRAS WT according to ERα and ERβ expression. **(H)** XY scatter plot of the mRNA levels of ESR1 (ERα), ESR2 (ERβ), and *KRAS* mutations from the TCGA-COAD database with 62 CRC patients. The data are presented as the mean ± SEM **(C,F)** or as the percentage **(E,G)**. The scale bar is 50 μm **(A–C)** and 100 μm **(F)**. *P <0.05, **P <0.01, ***P <0.001, paired t-test **(B)**, Mann-Whitney test **(F)** and χ_2_ test **(D,G)**.

We had 306 primary CRC samples available for the evaluation of ERα and ERβ expression. Fourteen patients, who were previously operated and treated for breast cancer, were excluded from the study due to the risk of ERα alterations from the anti-estrogen therapies (Figure 1). We successfully evaluated ERβ in 300 CRC patients and ERα in 270 CRC patients. Based on the staining intensity assessed with IHC, ERβ expression was categorized as low and high, while ERα expression was categorized as negative and positive (Figure 2A). We next compared the expression of these receptors between normal and matched cancer tissues and found that compared to ERα expression levels, ERβ expression levels were higher in both normal and cancer tissues (Figure 2B). However, compared to normal tissues, a downregulation of ERβ and an upregulation of ERα were observed in the matched CRC tissues (Figure 2B, see violin bar graph). Since we previously reported that high ERβ expression correlated with better prognosis in CRC (7), we investigated the distribution of ERα expression in patients with low and high ERβ expression. We grouped the patients into four categories based on ERα and ERβ expression (Figure 2C). We found that 79% of patients with high ERβ expression had also negative ERα expression compared with 63% in the low ERβ group (Figure 2D). Likewise, the percentage of patients with positive ERα expression was higher in the low ERβ expression group (37%) than in the high ERβ expression group (21%) (Figure 2D). For representative IHC images of matched pairs of patients for both ERα and ERβ expression, see Supplementary Figure 1A.

Next, we used *ESR1* (ERα) and *ESR2* (ERβ) mRNA levels from the TCGA-COAD database to investigate the differential expression of ERα and ERβ in CRC patients with TNM stage I disease and TNM stage IIIc+IV disease. Compared to those with stage I disease, a smaller percentage of patients with stage IIIc+IV disease had upregulated *ESR2* mRNA levels (Figure 2E). Additionally, *ESR2* levels were lower in patients with stage IIIc+IV disease than in those with stage I disease (Figure 2E). Furthermore, *ESR1* mRNA levels were obviously higher in patients with stage IIIc+IV disease than in those with stage I disease (Figure 2E).

### The Specificity of the ERα Antibody

Because the role of ERα expression in CRC is very little studied and all our results are based on antibody staining, we tested the specificity of the antibodies we used, in order to validate the antibodies. First, we stained the normal breast tissue, which is known to abundantly express ERα (positive control), and normal kidney, prostate, and skin tissues, which are known to lack ERα expression (negative controls, Supplementary Figure 1B) (25–27). Next, the same tissues were also stained with another anti-ERα antibody, D12 (Supplementary Figure 1C), which is widely used for the detection of ERα expression (28–30). We randomly stained 59 patients from the Female cohort with the D12 antibody. As shown in Supplementary Figure 1D the distribution of the IRS for nuclear ERα expression for each patient (*n* = 59) was the same for both antibodies. Likewise, when the patients were grouped as positive and negative nuclear ERα expression, no significant difference was observed between the two antibodies (*P* = 0.11, Supplementary Figure 1E). Out of 59 patients randomly stained with D12 antibody, 13 patients (22%) were positive for ERα expression, while 19 patients (32%) were detected as positive using the cocktail antibody (Supplementary Figure 1E). This could be explained by the fact that the cocktail antibody 1D5 + 6F11 was created by mixing two monoclonal antibodies that detect two different epitopes (31, 32). Representative IHC images of matched-pair CRC tissues for both antibodies are shown in the Supplementary Figure 1F.

### Correlation of ERα and ERβ Expression With KRAS Mutation Status

Out of 252 patients with successful staining for the KRAS mutation, only 31 (12.3%) had positive staining (Figure 2F). Patients with a KRAS mutation had a significantly higher intensity of ERα expression (*P* < 0.05) and a tendency to have lower ERβ expression (P = 0.06) than patients with wild-type (WT) KRAS (Figure 2F). Additionally, we observed that 19% of patients with positive ERα expression had KRAS mutations, while 9% of patients with negative ERα expression had KRAS mutations (Figure 2G). An opposite tendency was observed when looking at the distribution of KRAS mutations in patients with low and high ERβ expression. While 15% of patients with low ERβ expression had KRAS mutations, only 7% of patients with high ERβ expression had KRAS mutations (Figure 1G). However, no statistical significance was reached. To further validate these findings, we used mRNA data from the TCGA-COAD public database and found a strong and significant positive correlation between the mRNA levels of *ESR1* (ERα) and *KRAS* mutations, while no correlation was found with *ESR2* mRNA levels (ERβ) (Figure 2H).

### Evaluation of the Prognostic Relevance of ERα and ERβ Expression in CRC Patients

**Figure 3:**
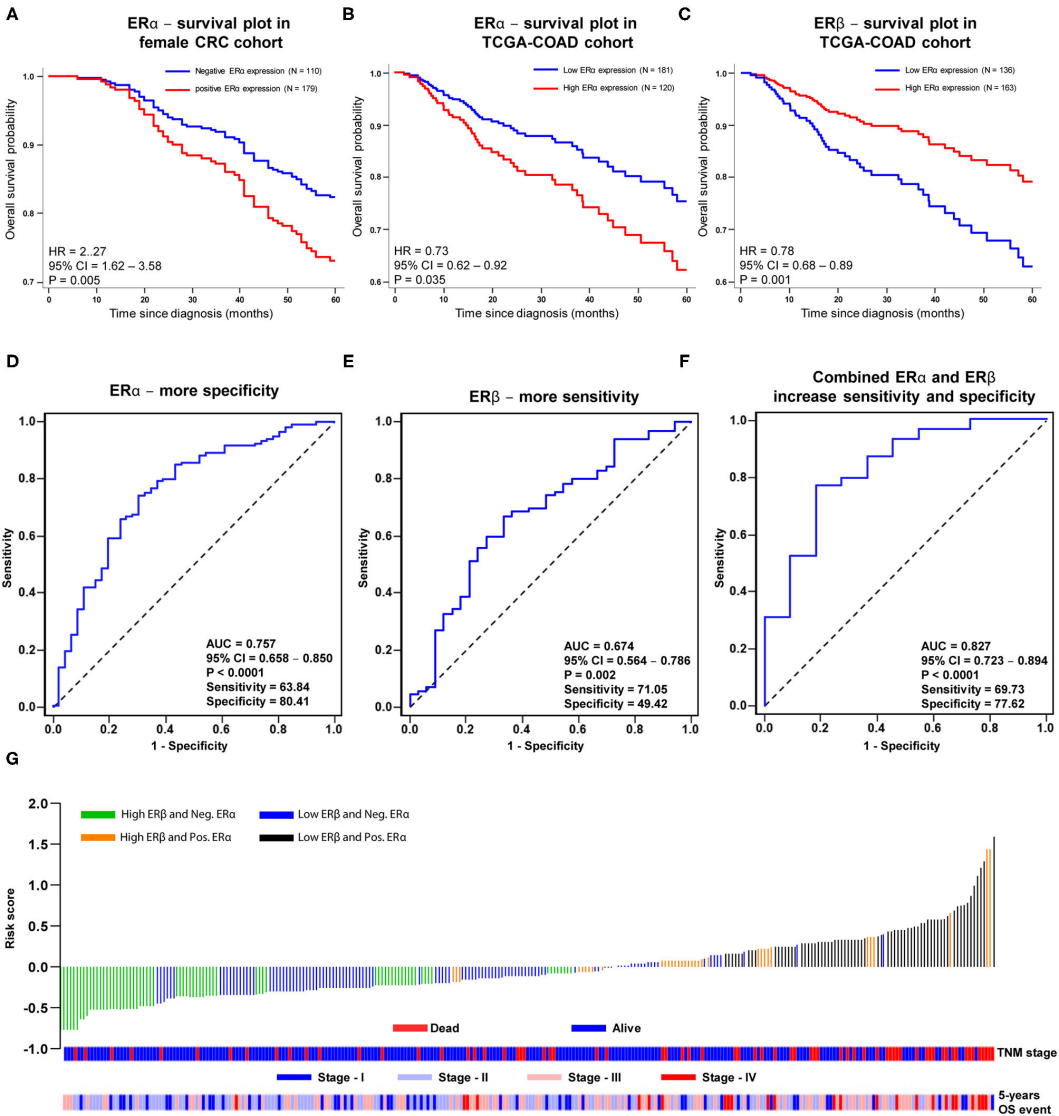
Prognostic assessment with sensitivity and specificity estimation for only ERα, ERβ and combined ERα – ERβ protein expression without clinical factors in female CRC and TCGA-COAD cohorts. Kaplan-Meier survival curves for: **(A)** ERα expression in female CRC cohort, **(B)** ERα and **(C)** ERβ expressions in TCGA-COAD cohort with cancer stage I-III. ROC curve, sensitivity and specificity analysis for the univariate model for **(D)** ERα, **(E)** ERβ and **(F)** combined ERα – ERβ protein expressions in female CRC cohort for 5-years OS. **(G)** Water fall plot for estimated risk score profile for combined ERα - ERβ protein expressions in four patients'groups in female CRC cohort with stage and event information (cutoff based on Youden's index association criteria with OS). *P*-values according to the log-rank test.

Previously we reported that high nuclear ERβ expression is independently associated with better OS and DFS in female CRC patients (7). Herein, we report that CRC patients with negative nuclear ERα expression have 19% lower risk for 5-years overall mortality (HR = 0.81; 95% CI, 0.68-0.94; *P* = 0.042, Figure 3A). Likewise, in the TCGA-COAD cohort, low ERα protein expression (HR = 0.73; 95% CI, 0.62-0.92; *P* = 0.035, Figure 3B) and high ERβ protein expression (HR = 0.78; 95% CI, 0.68-0.89; *P* = 0.001, Figure 3C) are associated with better prognosis of CRC patients. Additionally, we investigated the predicting ability of ERα and ERβ expression in our female patient's cohort calculating the ROC curves. We found that ERα expression predicts the 5-years OS with higher specificity (AUC = 0.720, Sensitivity = 65.22 and Specificity = 79.37, Figure 3D), while ERβ expression with higher sensitivity (AUC = 0.674, Sensitivity = 71.05 and Specificity = 49.42, Figure 3E). When we combined the ERα and ERβ expression, the predicting ability for 5-years OS in CRC patients was significantly improved with higher sensitivity and higher specificity (AUC = 0.842, Sensitivity = 71.53 and Specificity = 82.90, Figure 3F). Next, we looked at the risk score profile with TNM-stage and 5-years OS event by combining the ERα and ERβ expression in four groups as described above (Figure 2C). As shown in Figure 3G, the subgroups with positive ERα expression had the highest risk score profile, while the patients with negative ERα expression had the lowest risk score profile, despite the ERβ expression levels.

### Association of Combined ERα and ERβ Expression With OS and DFS in CRC Patients

**Figure 4:**
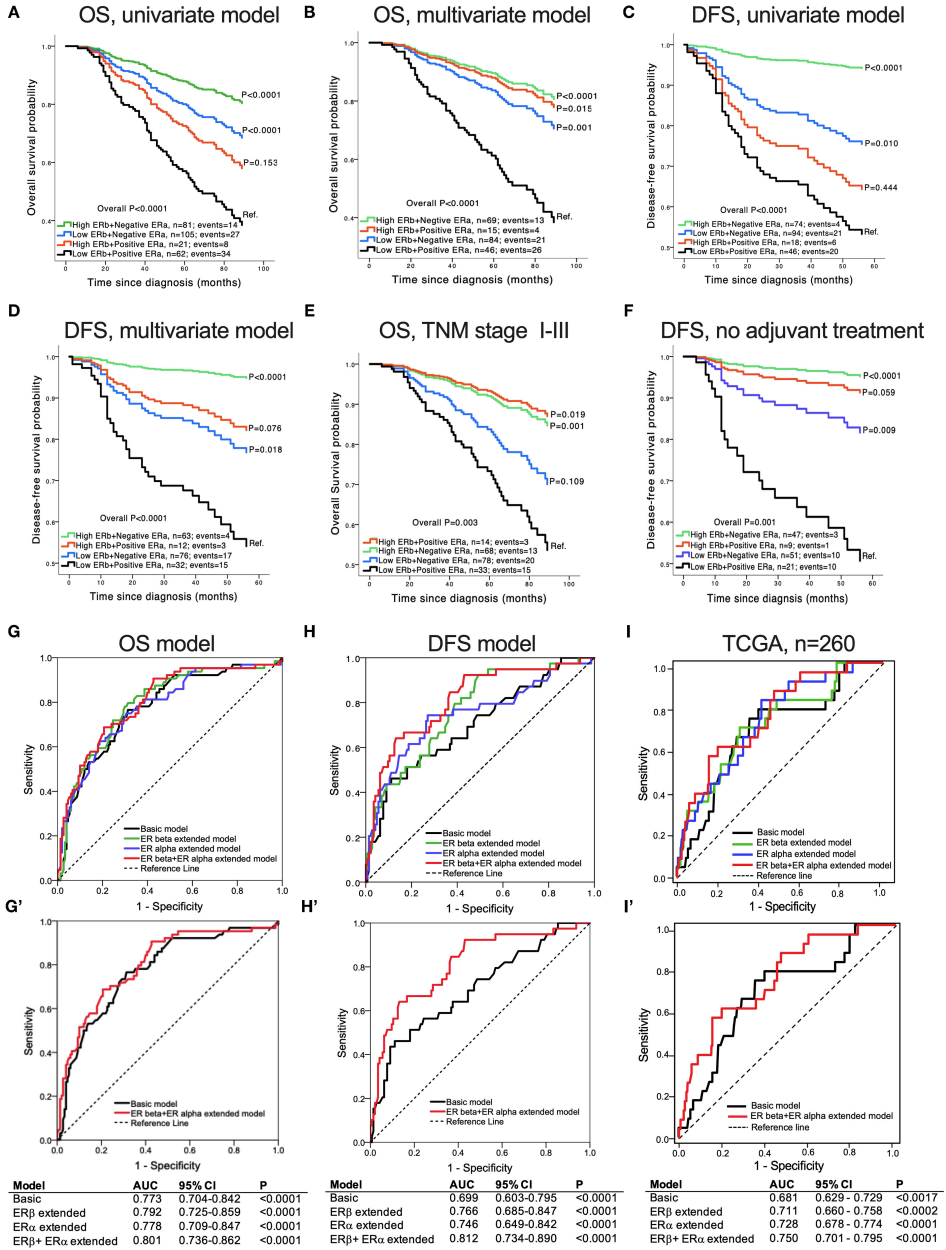
Association of concomitant ERβ and ERα expression with CRC patient survival. Kaplan-Meier survival curves for OS: **(A)** univariate model, *n* = 269; **(B)** multivariate model adjusted for age, TNM stage and tumor vascular invasion, *n* = 214; **(C)** multivariate model for patients with stage I-III cancer, *n* = 180. Kaplan-Meier survival curves for DFS: **(D)** univariate model, *n* = 232; **(E)** multivariate model adjusted for age, TNM stage and tumor vascular invasion, *n* = 183; **(F)** multivariate model for patients who did not receive adjuvant treatment after surgery, n =128. **(G–I)** ROC curves comparing the basic model (adjusted for age, TNM stage and tumor vascular invasion), the extended model including only ERβ expression, the extended model including only ERα expression, and the extended model with combined ERβ and ERα expression for OS **(G)** and DFS **(H)**. **(I)** ROC curves from the TCGA-COAD database for stage I-III colon cancer, comparing the basic model (adjusted for age, TNM stage and tumor vascular invasion), the extended model including only ERβ expression, the extended model including only ERα expression, and the extended model with combined ERβ + ERα expression for DFS**. (G'–I')** ROC curves comparing the basic model with the model including the combined ERβ and ERα protein expression for OS **(G')**, DFS **(H')** and DFS from the TCGA-COAD database **(I')**. The tables show the values of the area under the curve (AUC) for each of the corresponding models. *P*-values according to the log-rank test.

Next, we investigated the combined role of ERα and ERβ expression in CRC OS and DFS (Figure 4). The Cox regression analysis showed that patients with combined high ERβ + negative ERα expression were independently associated with better OS and had a 77% reduction in overall mortality (Figures 4A,B, Supplementary Table 1), as well as better DFS with a 90% reduction in cancer recurrence (Figures 4C,D, Supplementary Table 1) after adjustment for age, TNM stage and tumor vascular invasion, compared to patients with combined low ERβ + positive ERα expression, which were taken as the reference group. This finding was consistent even for the subgroups of patients with stage I-III cancer (Figure 4E), patients with colon cancer (Supplementary Figures 2A,B) and patients who did not receive adjuvant treatment (Figure 4F and Supplementary Figure 2C). In the second group of patients with low ERβ expression, even though the expression of ERα remained negative, the risk was increased by 14% for overall mortality and 33% for cancer recurrence compared to patients with combined high ERβ negative ERα expression (Supplementary Table 1). In addition, in the third group of patients with positive ERα expression, even though the expression of ERβ was high, the increase in the risks of overall mortality and cancer recurrence was much lower than that in the first group with combined high ERβ + negative ERα expression (3 and 22% lower, respectively: Supplementary Table 1, multivariate analysis). It is difficult to draw any conclusions about the subgroup of patients with rectal cancer due to the very small number of patients in each category, especially the category with combined high ERβ + positive ERα expression that has only one patient, *n* = 1 (Supplementary Table 1, Supplementary Figures 2D,E). These results clearly show that CRC patients with combined high ERβ + negative ERα expression have the best prognosis and that the subgroup with combined low ERβ + positive ERα expression has the worst prognosis.

### Predictive Ability of Combined ERα and ERβ Expression

To further investigate the role of the combined ERα and ERβ expressions in predicting CRC prognosis, we evaluated the ROC curves for the basic model (adjusted for age, TNM stage and tumor vascular invasion), the model extended with only ERβ expression, the model extended with only ERα expression, and the model that included the combined ERβ + ERα expressions. As shown in Figures 4G,H, the AUC was significantly higher for the model with the combined ERβ + ERα expressions than for all the other models for both OS and DFS. However, the predictive ability of the combined ERβ + ERα extended model was higher for DFS (AUC = 0.812, Figure 4H') than for OS (AUC = 0.801, Figure 4G'). The same results were obtained using the TCGA-COAD external cohort, where the combined expression of ERs had the best predictive ability for DFS compared with the other models (Figures 4I,I'). These results clearly show that the combined expression of ERα and ERβ plays an important role in predicting the prognosis of CRC patients.

### Distribution of Clinical Parameters and Tumor Characteristics in Patients With Combined High ERβ + Negative ERα Expression VS. Patients With Combined Low ERβ + Positive ERα Expression

We aimed to evaluate the distribution of clinical parameters and tumor characteristics between patients with combined high ERβ + negative ERα expression, considered to be the best prognostic group, and those with combined low ERβ + positive ERα expression, considered to be the worst prognostic group. As shown in Table 1, patients with combined high ERβ + negative ERα expression had a significantly lower number of overall deaths and cancer recurrence events, smaller tumor extent, fewer tumor metastases in the regional lymph nodes and distant organs, predominantly stage I and II disease, and were less likely to receive adjuvant treatment after the operation. Additionally, tumors with combined high ERβ + negative ERα expression had a higher frequency of the mucinous type of COAD and a never smoking status (Table 1).

**Table 1:** Distribution of clinical parameters and tumor characteristics in 143 CRC patients according to subgroups with combined high ER&-negative ERa and combined low ERB-positive ERa expressions.

	**Total**	**High ERß** **Negative ERα**	**Low ERß** **Positive ERα**	
**Characteristics**	**N (%)**	**N (%)**	**N (%)**	***P***
Patients no.	143 (100)	81 (56)	62 (44)	
Deaths	48 (34)	14 (29)	34 (71)	<0.0001^a^
DFS events^*^	24 (19)	4 (17)	20 (83)	<0.0001^a^
Age (mean, years)	70.9	71.8	69.8	0.198^b^
BMI (mean, kg/m^2^)	26.1	25.9	26.2	0.931^b^
Tumor extent	41 (29	30 (73)	11 (27)	0.011^a^
≤T2	102	51 (50)	51 (50)	
>T2	(71)			
Lymph node metastasis	90 (63)	60 (67)	30 (33)	0.002^a^
N0	53 (37)	21 (40)	32 (60)	
N1/N2				
Distant metastasis at diagnosis	128 (89)	80 (63)	48 (37)	<0.0001^a^
M0	15 (11)	1 (7)	14 (93)	
M1				
TNM stage	30 (21)	21 (70)	9 (30)	<0.0001^a^
I	55 (39)	38 (69)	17 (31)	
II	42 (29)	20 (48)	22 (52)	
III	15 (11)	1 (7)	14 (93)	
IV	1			
Missing				
Tumor intravascular invasion	83 (72)	53 (64)	30 (36)	0.174^a^
No	32 (28)	16 (50)	16 (50)	
Yes	28			
Missing				
Tumor differentiation	21 (15)	14 (67)	7 (33)	0.354^a^
Low	120	67 (56)	53 (44)	
Moderate/High	(85) 2			
Missing				
Tumor localization	106 (74)	58 (55)	48 (45)	0.431^a^
Colon	37 (26)	23 (62)	14 (38)	
Rectum				
Tumor histological type	110 (77)	57 (52)	53 (48)	0.079^a^
Non-mucinous AC^†^	22 (15)	15 68)	7 (32)	
Partly Mucinous AC	11 (8)	9 (82)	2 (18)	
Mucinous AC				
Neoadjuvant treatment	124 (87)	70 (57)	54 (43)	0.906^a^
No	19 (13)	11 (58)	8 (42)	
Yes				
Adjuvant treatment	99 (71)	63 (64)	36 (36)	0.016^a^
No	41 (29)	17 (42)	24 (58)	
Yes	3			
Missing				
Smoking status	5 (11)	1 (20)	4 (80)	0.059^a^
Ever smokers	39 (89)	25 (64)	14 (36)	
Never smokers	99			
Missing				
Alcohol use	19 (43)	9 (47)	10 (53)	0.168^a^
Yes	25 (57)	17 (68)	8 (32)	
No	99			
Missing				

* *Patients with TNM stage IV are excluded*.

a *Pearson chi-square test*.

b *Mann-Whitney U test*.

† *AC, Adenocarcinoma; BMI, Body Mass Index*.

### Correlation of Combined ERα and ERβ Expression With Hormonal Characteristics in Female Patients With CRC

**Figure 5:**
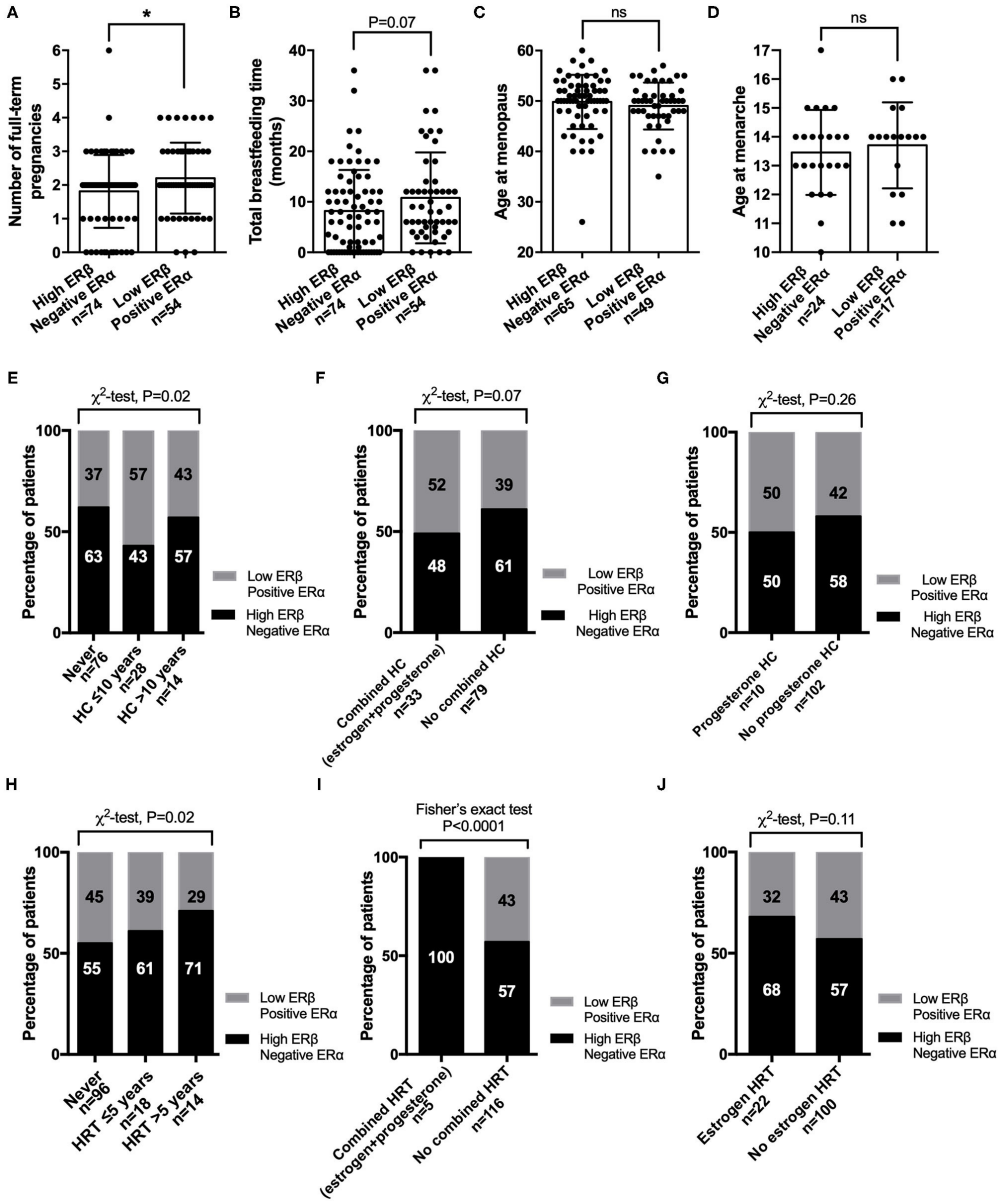
Correlation of hormonal status with subgroups of female CRC patients with both ERβ and ERα expression. Hormonal characteristics for **(A)** number of full-term pregnancies, where 0 refers to women who never had children; **(B)** total breastfeeding time for all the children a woman had, where 0 refers to women who never breastfed; **(C)** age at menopause; and **(D)** age at menarche. Percentage of female CRC patients with combined high ERβ + negative ERα expression or combined low ERβ + positive ERα expression who never or ever used **(E)** hormonal contraception (HC); **(F)** combined (estrogen and progesterone) HC; **(G)** progesterone HC; **(H)** hormonal replacement therapy (HRT); **(I)** combined (estrogen and progesterone) HRT; or **(J)** estrogen HRT. The data are presented as the mean ± SEM **(A–D)**. ^*^P <0.05, unpaired *t*-test; χ_2_ test or Fisher's exact test as indicated.

We explored the hormonal characteristics of CRC female patients in relation to the combined ERα and ERβ expression. We found that female patients with combined high ERβ + negative ERα expression had a lower number of pregnancies (mean ± standard error of the mean, 1.8 ± 0.13, *P* = 0.04; Figure 5A) and shorter breastfeeding times (calculated as the total breastfeeding months for all the children a woman had; 8.2 ± 0.95, *P* = 0.08; Figure 5B) than female patients with combined low ERβ + positive ERα expression (2.2 ± 0.14 and 10.8 ± 1.2, respectively). No significant differences were observed between the two groups regarding the age of menopause and age of menarche (Figures 5C,D). Next, we examined how the use of hormonal contraception (HC) differed between the two groups. We found that most of the female patients with combined high ERβ + negative ERα expression never used HC compared with women with combined low ERβ + positive ERα expression (63% vs. 37%, *P* = 0.02, Figure 5E). When we looked at the type of HC, we found that 61% of female patients with combined high ERβ + negative ERα expression had never used combined (estrogen and progesterone) HC and 48% of them had used combined HC. In the subgroup of women with combined low ERβ + positive ERα expression 39% had never used combined HC and 52% had used combined HC (*P* = 0.07, Figure 5F). However, no difference was observed between the two groups regarding the use of progesterone HC (Figure 5G). We also looked at the use of hormone replacement therapy (HRT) and found that most of the female patients with combined high ERβ + negative ERα expression had used HRT for more than 5 years, while very few female patients with combined low ERβ + positive ERα expression had used HRT for a long time (71 and 29%, respectively, *P* = 0.02, Figure 5H). All the female patients who had used combined (estrogen and progesterone) HRT had combined high ERβ + negative ERα expression (*P* < 0.0001; Figure 5I). No significant results were found regarding the use of estrogen HRT (Figure 5J).

### Correlation of Combined ERα and ERβ Expression With Proteins Important for CRC Progression and Development

**Figure 6:**
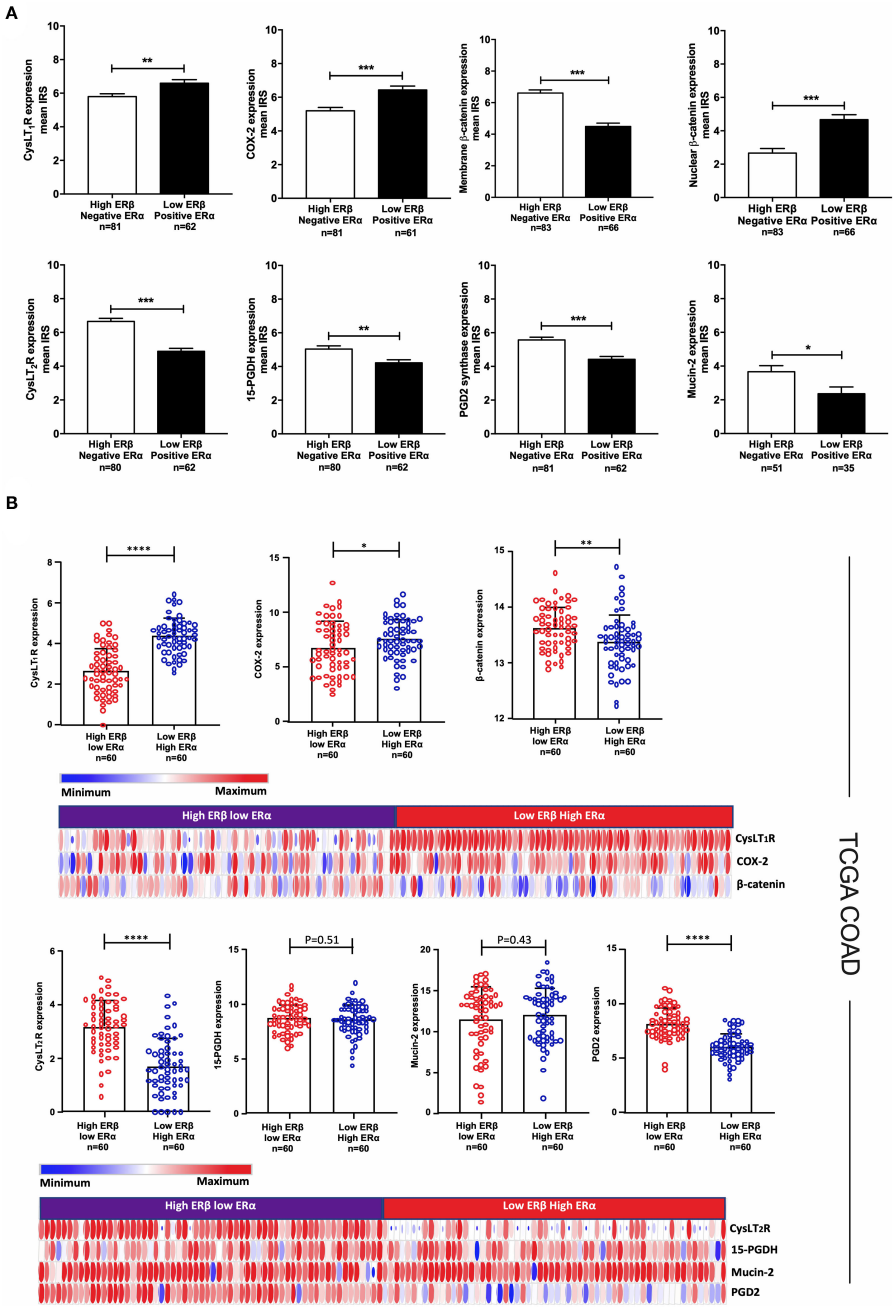
Correlation of subgroups of patients with ERβ and ERα expression with proteins important for CRC progression and development. **(A)** Mean IRS for CysLT_1_R, COX-2, membrane and nuclear β-catenin, CysLT_2_R, 15-PGDH, Mucin-2, and PGD2 synthase expression levels evaluated with IHC in subgroups of CRC patients with combined high ERβ + negative ERα expression (*n* = 81) or combined low ERβ + positive ERα expression (*n* = 62). **(B)** Expression of the indicated proteins (CysLT_1_R, COX-2, β-catenin, CysLT_2_R, 15-PGDH, Mucin-2 and PGD2 synthase) in the TCGA-COAD patients with combined high ERβ + low ERα expression (*n* = 60) or combined low ERβ + high ERα expression (*n* = 60) together with the corresponding heat maps. The data are presented as the mean ± SEM. ^*^P < 0.05, ^**^P < 0.01, ^***^P < 0.001, ^****^P < 0.0001, Mann-Whitney test.

To further explore the prognostic role of combined ERα and ERβ expression in CRC patients, we correlated the patient with combined high ERβ + negative ERα expression or combined low ERβ + positive ERα expression with proteins important in CRC development and progression (Figure 6A). We noticed that patients with combined low ERβ + positive ERα expression had lower IRSs for CysLT_1_R (*P* < 0.01), COX-2 (*P* < 0.001) and nuclear β-catenin (*P* < 0.001), which are connected to enhanced cell proliferation and poor patient outcome (18, 19, 23), compared to patients with combined high ERβ + negative ERα expression (Figure 6A, Supplementary Figure 3 for IHC images). On the other hand, patients with combined high ERβ + negative ERα expression had higher IRSs for CysLT_2_R (*P* < 0.001), membrane β-catenin (*P* < 0.001), 15-PGDH (*P* < 0.01) and PGD2 synthase (*P* < 0.001), which are associated with a better outcome in CRC (20, 23, 33, 34) (Figure 6A, Supplementary Figure 3). Since we observed a higher frequency of mucinous adenocarcinomas in the group of patients with combined high ERβ + negative ERα expression, we investigated the association with Mucin-2 expression known to be reduced in CRC tissues compared to the normal mucosa (35, 36). We found that patients with combined high ERβ + negative ERα expression had significantly higher IRSs for Mucin-2 expression levels (*P* < 0.05) than patients with combined low ERβ + positive ERα expression (Figure 6A, Supplementary Figure 3). In the TCGA-COAD cohort, the same correlations were observed between the combined protein expression of ERs and CysLT_1_R, COX-2, CysLT_2_R and PGD2 synthase, whereas no correlation was found for combined ERs expression with 15-PGDH and Mucin-2 expression levels (Figure 6B).

### Association of ERα and ERβ Expression With Metastasis in Patients With CRC

**Figure 7:**
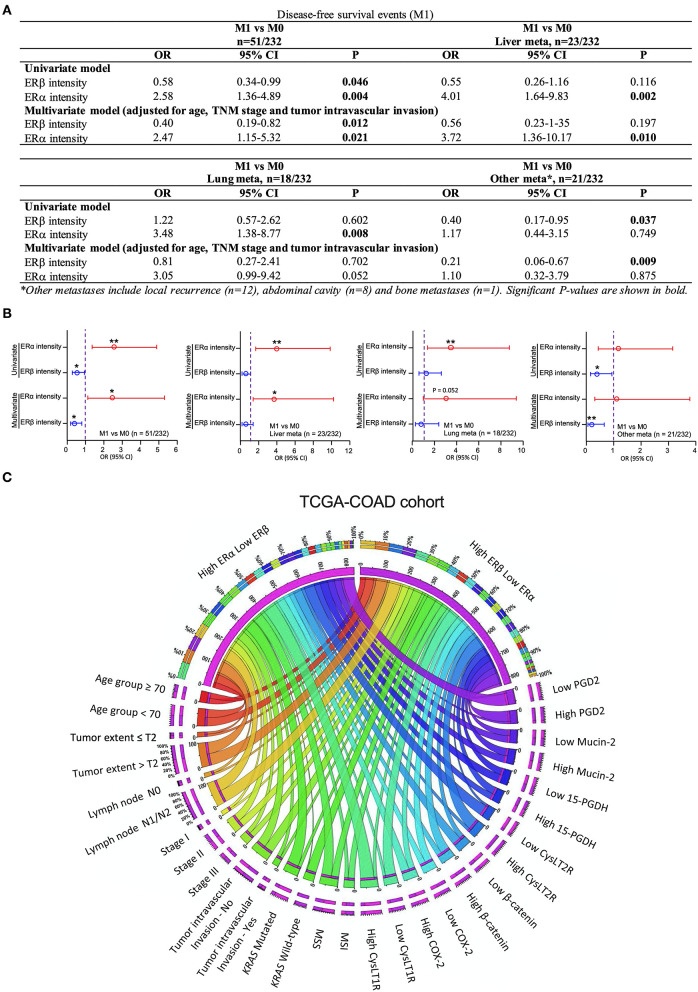
Correlation of ERβ and ERα expression with CRC metastasis. **(A)** Binary logistic regression model showing the odds ratios (ORs) and 95% confidence intervals (CIs) for total metastatic events; liver metastasis; lung metastasis; other metastases; ocal recurrences; abdominal metastasis and bone metastasis. **(B)** Forest plots showing the respective estimates for the corresponding metastatic events for the patients included in the study. **(C)** Distributions of each clinical factor and associated protein expression pattern in the combined high ERβ + low ERα or combined low ERβ + high ERα expression groups in the TCGA-COAD cohort. The data were visualized *via* Circos software. The area of each colored ribbon depicts the frequency of the samples. *P < 0.05, **P < 0.01.

We investigated the risk of having a metastatic event for each unit increase in the ERβ and ERα staining intensity, evaluated by IHC. We found that for each unit increase in the ERβ intensity, the risk of having a metastatic event were significantly and independently decreased by 60% after adjustment for age, TNM stage and tumor vascular invasion (OR = 0.40; 95% CI: 0.19–0.82; *P* = 0.012; Figure 7A). In addition, for each unit increase in the ERα intensity, the risk of having a metastatic event increased almost 2.5-fold (OR = 2.47; 95% CI: 1.15–5.32; *P* = 0.021; Figures 7A,B). The ERα intensity was strongly associated with liver metastasis, where for each unit increase in the ERα intensity, the risk of liver metastasis independently increased almost 4-fold (OR = 3.72; 95% CI: 1.36–10.17; *P* = 0.01; Figures 7A,B). However, no role of ERβ was found in lung metastasis and the promoting effect of increased ERα staining intensity (OR = 3.48; 95% CI: 1.38–8.77; *P* = 0.008) disappeared after adjustment for other confounding factors (OR = 3.05; 95% CI: 0.99–9.42; *P* = 0.052; Figure 7A). Importantly, each unit increase in the ERβ intensity significantly and independently decreased the risk of local recurrence and abdominal metastasis by 79% (OR = 0.21; 95% CI: 0.06–0.67; *P* = 0.009; Figures 7A,B). These results were summarized graphically using the forest plots, where the increased risk is shown in red, and the decreased risk is shown in blue (Figure 7B).

## Discussion

CRC is one of the most common malignancies worldwide. Despite the current technologies for early detection and targeted therapies, the risk of recurrence in patients with stage II and III cancer remains high (37). Prognostic markers are needed to predict the recurrence risk with higher precision. Herein, we demonstrate the prognostic significance of the combined ERα and ERβ expression in female patients with CRC and explore their correlations with other prognostic markers and hormonal status.

We found that in cancer tissues, ERβ expression was downregulated while ERα expression upregulated, compared to the normal matched pair tissues (Figure 2B). We previously reported that high ERβ expression is associated with better OS and DFS (7), and in this investigation we showed that most of the patients with high ERβ expression were negative for ERα expression, while the majority of patients with low ERβ expression were positive for ERα expression. Many have reported the downregulation of ERβ during tumor progression (2–4, 7), while others have shown that ERα protein levels significantly increase in men but not in women with CRC (38). Herein, we showed that ERα expression levels are increased in cancer tissues compared to matched normal tissues in females with CRC. A previous report detected ERα and ERβ protein levels in CRC and they found no significant difference of ERβ expression levels between normal and cancer colon tissues (39). Another report showed that ERα expression is rare in CRC tissue and its expression does not correlate with colon carcinogenesis, while ERβ expression was upregulated in CRC tissues and correlated with poor DFS (40). It is worth noting that both studies had a small number of patients and included in their studies even colon adenomas (41). Moreover, both studies used polyclonal antibodies and the antibody used from Grivas et at., recognizes only the β1 isoform (40).

Furthermore, we investigated the correlation of ERα and ERβ expression with KRAS mutation, which plays an important role in the prognosis and treatment of CRC (15). In 4,411 CRC patients, *KRAS* mutations were independently associated with shorter relapse times, survival after recurrence and OS in patients with MSS but not MSI tumors (16). Additionally, treatment with anti-EGFR is ineffective in CRC patients with *KRAS* mutations (17). Interestingly, we found that patients with positive ERα expression, which were associated with shorter OS (Figures 3A,B), had a higher frequency of KRAS mutations than patients with negative ERα expression. This result was further supported by mRNA data from the TCGA-COAD cohort, where we found a significant positive correlation between the mRNA levels of *ESR1* (ERα) and *KRAS* mutations. This finding can provide new opportunities for patients with *KRAS* mutations, where ERα-selective antagonists might be an alternative to improve their prognosis. No correlations were observed between KRAS status and ERβ expression at either expression level detected by IHC or mRNA levels from the TCGA-COAD cohort.

Next, we evaluated the prognostic role of the combined ERα and ERβ expression in CRC patient survival. Patients with combined high ERβ + negative ERα expression had the best OS and DFS, with a reduction in overall mortality by 77% and cancer recurrence by 90%. Patients with combined low ERβ + positive ERα expression, taken as the reference category, had the worst OS and DFS. The model with the combined expression of ERs had the highest predicting ability compared to all the other models taken into consideration. Moreover, we found that each unit increase in the ERα intensity independently increased the risk of liver metastasis almost 4-fold, while each unit increase in the ERβ intensity reduced the risk of local recurrence and abdominal metastasis by 79%. These results imply an important role of the combined ERα and ERβ expression as a future prognostic marker in patients with CRC. Reports show that CysLT_1_R, CysLT_2_R, COX-2 and β-catenin expression levels are linked to CRC development and prognosis (42). High levels of 15-PGDH and PGD2 synthase in CRC are reported to have antitumor properties (20–22, 33, 34). We found that patients with combined high ERβ + negative ERα expression had significantly lower IRSs of tumor-promoting proteins, such as CysLT_1_R, COX-2 and nuclear β-catenin, and higher IRSs of anti-tumorigenic proteins such as CysLT_2_R, membrane β-catenin, 15-PGDH and PGD2 synthase, compared to patients with combined low ERβ + positive ERα expression. To validate our findings, we used protein data from the TCGA-COAD cohort and found that compared to patients with combined low ERβ + high ERα expression, patients with combined high ERβ + low ERα expression had a better tumor profile and a more favorable prognosis (Figure 7C).

Interestingly, we found that patients with combined high ERβ + negative ERα expression had significantly smaller tumors, fewer regional and distant metastases, predominantly TNM stage I and II and were less likely to receive adjuvant treatment. In addition, patients with combined high ERβ + negative ERα expression were more likely to have a never smoking status, which is an established risk factor for CRC (43), and a higher frequency of mucinous adenocarcinoma, which also correlated with higher IRS for Mucin-2 expression. High Mucin-2 levels are linked to colon cell differentiation (36, 44). Previous studies have shown that ERs are implicated in the obesity-associated CRC (12, 13), however we found no correlation between BMI and the combined ERα and β expression.

We previously found that high ERβ expression in female CRC patients was associated with a lower number of pregnancies, shorter breastfeeding times, a longer time of combined HC use, and a longer time of HRT use (7). Many studies have suggested a lower risk of CRC incidence among women who use HRT (45). However, none of them took into consideration the combined expression of ERα and ERβ in CRC tissue. Herein, we showed that in female CRC patients, combined high ERβ + negative ERα expression correlated with lower pregnancy number, shorter breastfeeding times, non-use of HC and long-term use of HRT, both estrogen monotherapy and combined HRT.

An important issue to address is the antibody used in IHC. The use of TMAs in cancer research raises the concern whether the chosen core tissue is representative of the whole tumor. However, the use of two cores to represent the tumor has shown sufficient concordance for many cancer types, including CRC (46). The clone 14C8 of the anti-ERβ antibody that we used, recognizes most of ERβ variants including ERβ wild-type, and is shown to be useful for the assessment of ERβ expression in paraffin-embedded tissues (47). In a recent publication for the validation of ERβ antibodies in 44 different tissues, 14C8 antibody showed in CRC IHC the same intensity band as PPZ0506, which was reported to be the most specific anti- ERβ antibody, and that correlated with ERβ mRNA levels detected in the CRC tissue [Figure 3, see reference (48)]. Because ERα is low expressed in the colon tissue, we used a cocktail antibody (1D5 + 6F11) created by mixing two monoclonal antibodies that target ERα. Human normal tissues verified for ERα expression levels were used as positive and negative controls to test the antibody specificity (25–27). To validate the IHC staining, 59 randomly selected patients from the cohort were stained with another ERα monoclonal antibody D12, widely used for the detection of ERα (28–30). The same control tissues that were stained positive for ERα expression using the cocktail antibody, were also stained positive with D12 antibody but the staining intensity was weaker. This was the reason that we identified more patients with positive ERα expression using the cocktail antibody, which might be missed using the monoclonal D12 antibody (32). It is important to highlight that we validated our findings by using protein expression data from the TCGA-COAD cohort, which was used as an external cohort and includes both female and male patients.

To the best of our knowledge, this is the first study to investigate the prognostic significance of combined ERα and ERβ expressions in CRC patients. Our results suggest that patients with combined high ERβ + negative ERα expression have a better outcome with longer OS and DFS. Interestingly, ERβ intensity was important for the local recurrence of CRC, while the ERα intensity was important for the liver metastasis. ERβ expression levels are found significantly decreased in CC tissues of both males and females compared to the matched normal mucosa, and ERα/ERβ protein ratio are altered in both male and female CRC tissues (38). Therefore, we believe that our results are applicable to both female and male CRC patients. In summary, our results highlight the role of combined expression of ERα and ERβ as important prognostic and treatment markers in CRC patients.

## Data Availability Statement

The datasets used and analyzed in the current study are available from the corresponding author upon request.

## Ethics Statement

The studies involving human participants were reviewed and approved by Lund University Ethical Committee Approval 3/2006. The patients/participants provided their written informed consent to participate in this study.

## Author Contributions

GT and AS: conception and design. GT, SG, and M-LL: development of methodology. GT, SG, RE, and AS: analysis and interpretation of data. RE and ML-L: administrative and/or material support. GT, AS, SG, and SS: writing and review of the manuscript. All authors have read, reviewed, and approved the final version of the manuscript.

## Funding

The study was supported by grants awarded to AS from the Malmo University Hospital Cancer Foundation (UMAS Cancer foundation), the Swedish Cancer Foundation (grant no. 18 0748), and the Swedish Research Council (grant no. 17 01274), government funding for clinical research from the National Health Services (ALF), and funding awarded to GT, SG, and SS from the Royal Physiographical Society in Lund.

## Conflict of Interest

The authors declare that the research was conducted in the absence of any commercial or financial relationships that could be construed as a potential conflict of interest.

## Publisher's Note

All claims expressed in this article are solely those of the authors and do not necessarily represent those of their affiliated organizations, or those of the publisher, the editors and the reviewers. Any product that may be evaluated in this article, or claim that may be made by its manufacturer, is not guaranteed or endorsed by the publisher.

## Abbreviations

ERβ: estrogen receptor beta
ERα: estrogen receptor alpha
CC: colon cancer
CRC: colorectal cancer
DFS: disease-free survival
OS: overall survival.
