# Supplementary material for: Combined Estrogen Alpha and Beta Receptor Expression Has a Prognostic Significance for Colorectal Cancer Patients
Source: Front Med (Lausanne). 2022 Mar 14;9:739620. doi: 10.3389/fmed.2022.739620 (PMC8963951; doi:10.3389/fmed.2022.739620)
Supplement: Supplementary file 1 [file Table_1.pdf]

Supplementary table 1. Association of ERβ and ERα expressions with overall and disease-free survival.

Variables	Overall survival HR (95% CI)	P	Disease-free survival* HR (95% CI)	P
Univariate model (n=269, events=83; DFS, n=232, events=51)				
High ERβ+Negative ERα	0.23 (0.12-0.43)	<0.0001	0.10 (0.03-0.28)	<0.0001
Low ERβ+Negative ERα	0.39 (0.24-0.66)	<0.0001	0.44 (0.24-0.82)	0.010
High ERβ+Positive ERα	0.57 (0.26-1.23)	0.153	0.70 (0.28-1.74)	0.444
Low ERβ+Positive ERα	1 (Reference)		1 (Reference)	
Multivariate model (n=269, effective n=214, events=64; DFS, n=232, effective n=183, events=39)				
High ERβ+Negative ERα	0.23 (0.11-0.45)	<0.0001	0.10 (0.03-0.26)	<0.0001
Low ERβ+Negative ERα	0.37 (0.20-0.66)	0.001	0.43 (0.21-0.86)	0.018
High ERβ+Positive ERα	0.26 (0.09-0.77)	0.015	0.32 (0.09-1.04)	0.076
Low ERβ+Positive ERα	1 (Reference)		1 (Reference)	
Variables included in the multivariate model				
Age	1.06 (1.03-1.09)	<0.0001	1.01 (0.98-1.04)	0.479
TNM stage				
II vs I	1.26 (0.51-3.08)	0.618	3.57 (0.81-15.7)	0.093
III vs I	1.36 (0.53-3.45)	0.520	5.48 (1.27-23.7)	0.023
IV vs I	3.03 (1.09-8.36)	0.032	NA	NA
Tumor intravascular invasion				
Yes vs No	2.16 (1.24-3.74)	0.006	3.77 (1.93-7.36)	<0.0001
Multivariate model, patients with colon cancer (n=204, effective n=167, events=52; DFS, n=174, effective n=142, events=29)				
High ERβ+Negative ERα	0.25 (0.12-0.54)	<0.0001	0.05 (0.01-0.25)	<0.0001
Low ERβ+Negative ERα	0.36 (0.19-0.71)	0.003	0.39 (0.18-0.87)	0.021
High ERβ+Positive ERα	0.21 (0.06-0.71)	0.012	0.23 (0.05-1.03)	0.054
Low ERβ+Positive ERα	1 (Reference)		1 (Reference)	
Multivariate model, patients with rectal cancer (n=65, effective n=47, events=12; DFS, n=58, effective n=41, events=10)				
High ERβ+Negative ERα	0.12 (0.02-0.77)	0.026	0.15 (0.02-1.24)	0.079
Low ERβ+Negative ERα	0.36 (0.08-1.55)	0.171	0.46 (0.07-3.02)	0.418
High ERβ+Positive ERα	2.90 (0.09-87.5)	0.540	NA	NA
Low ERβ+Positive ERα	1 (Reference)		1 (Reference)	
Multivariate model, patients with TNM stage I-III (n=247, effective n=193, events=51)				
High ERβ+Negative ERα	0.27 (0.13-0.57)	0.001	NA	NA
Low ERβ+Negative ERα	0.57 (0.29-1.13)	0.109		
High ERβ+Positive ERα	0.22 (0.06-0.78)	0.019		
Low ERβ+Positive ERα	1 (Reference)			
Multivariate model, patients who did not received adjuvant treatment (n=178, effective n=144, events=43; DFS, n=160, effective n= 128, events=24)				
High ERβ+Negative ERα	0.26 (0.12-0.59)	0.001	0.07 (0.02-0.28)	<0.0001
Low ERβ+Negative ERα	0.39 (0.18-0.84)	0.016	0.29 (0.12-0.74)	0.009
High ERβ+Positive ERα	0.16 (0.04-0.73)	0.017	0.13 (0.02-1.07)	0.059
Low ERβ+Positive ERα	1 (Reference)		1 (Reference)	

*Patients with cancer stage IV were excluded from the analysis. NA = non-applicable.
