# Supplementary material for: Combined Estrogen Alpha and Beta Receptor Expression Has a Prognostic Significance for Colorectal Cancer Patients
Source: Front Med (Lausanne). 2022 Mar 14;9:739620. doi: 10.3389/fmed.2022.739620 (PMC8963951; doi:10.3389/fmed.2022.739620)
Supplement: Supplementary file 2 [file Data_Sheet_1.pdf]

Supplementary figure 1 “Prognostic relevance of concomitant estrogen receptor beta and estrogen receptor alpha expression in female colorectal cancer patients”.

Supplementary figure 1: The specificity of the ERα cocktail (1D5+6F11) antibody.

(A) The representative IHC pictures for the matched-pair CRC tissues for ERβ and ERα expression. **(B)** The representative IHC pictures showing the nuclear ERα expression in the normal breast tissue (positive control) and normal kidney, prostate, and skin tissues (negative controls) stained with ERα cocktail (1D5+6F11) antibody. **(C)** The representative IHC pictures showing the nuclear ERα expression in the normal breast tissue (positive control) and normal kidney, prostate, and skin tissues (negative controls) stained with ERα D12 antibody. **(D)** The distribution of the IRS for nuclear ERα expression for each patient (n=59) for both cocktail (1D5+6F11) and D12 antibodies. **(E)** The percentage of patients (n=59) scored as negative and positive nuclear ERα expression using the cocktail (1D5+6F11) and D12 antibodies. **(F)** The representative IHC pictures for the matched-pair CRC tissues with negative and positive nuclear ERα expression using the cocktail (1D5+6F11) and D12 antibodies.

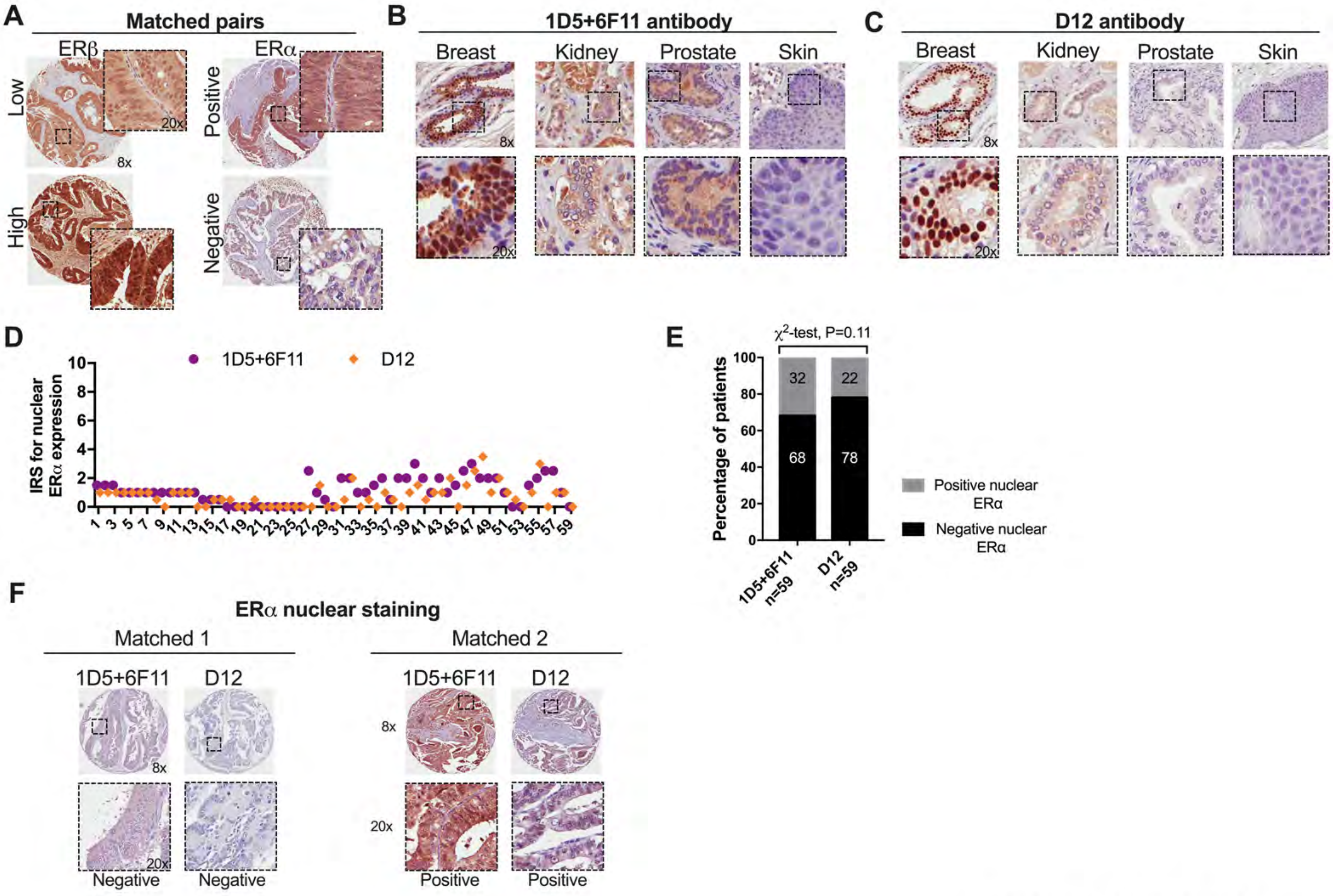

Supplementary figure 1
