# Supplementary material for: Combined Estrogen Alpha and Beta Receptor Expression Has a Prognostic Significance for Colorectal Cancer Patients
Source: Front Med (Lausanne). 2022 Mar 14;9:739620. doi: 10.3389/fmed.2022.739620 (PMC8963951; doi:10.3389/fmed.2022.739620)
Supplement: Supplementary file 3 [file Data_Sheet_2.pdf]

Supplementary figure 2 for the manuscript "Prognostic relevance of concomitant estrogen receptor beta and estrogen receptor alpha expression in female colorectal cancer patients".

Supplementary figure 2: Association of ERβ and ERα expression with CRC patient's survival.

Kaplan-Meier survival curves for OS **(A)** multivariate model for patients with colon cancer, n=167; **(B)** multivariate model for patients with rectal cancer, n=47; **(C)** multivariate model for patients that did not received adjuvant treatment after operation, n=144. Kaplan-Meier survival curves for DFS **(D)** multivariate model for patients with colon cancer, n=142; **(E)** multivariate model for patients with rec

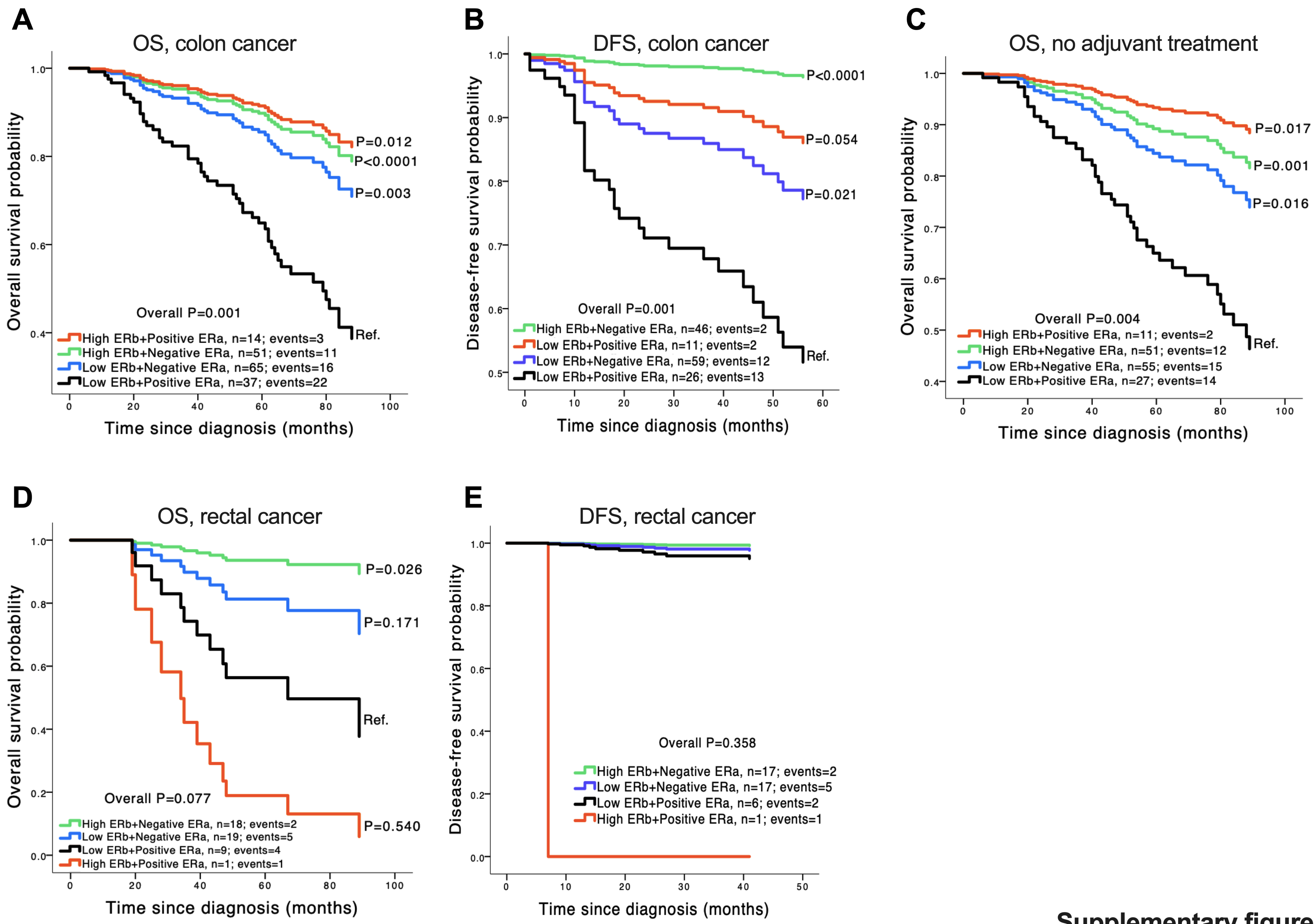

Supplementary figure 2
