# Supplementary material for: Combined Estrogen Alpha and Beta Receptor Expression Has a Prognostic Significance for Colorectal Cancer Patients
Source: Front Med (Lausanne). 2022 Mar 14;9:739620. doi: 10.3389/fmed.2022.739620 (PMC8963951; doi:10.3389/fmed.2022.739620)
Supplement: Supplementary file 4 [file Data_Sheet_3.pdf]

Supplementary figure 3 for the manuscript "Prognostic relevance of concomitant estrogen receptor beta and estrogen receptor alpha expression in female colorectal cancer patients".

Supplementary figure 3:

Representative microscopic IHC images showing the expression of indicated proteins at 8× and 40× magnification for the main image in figure 4.

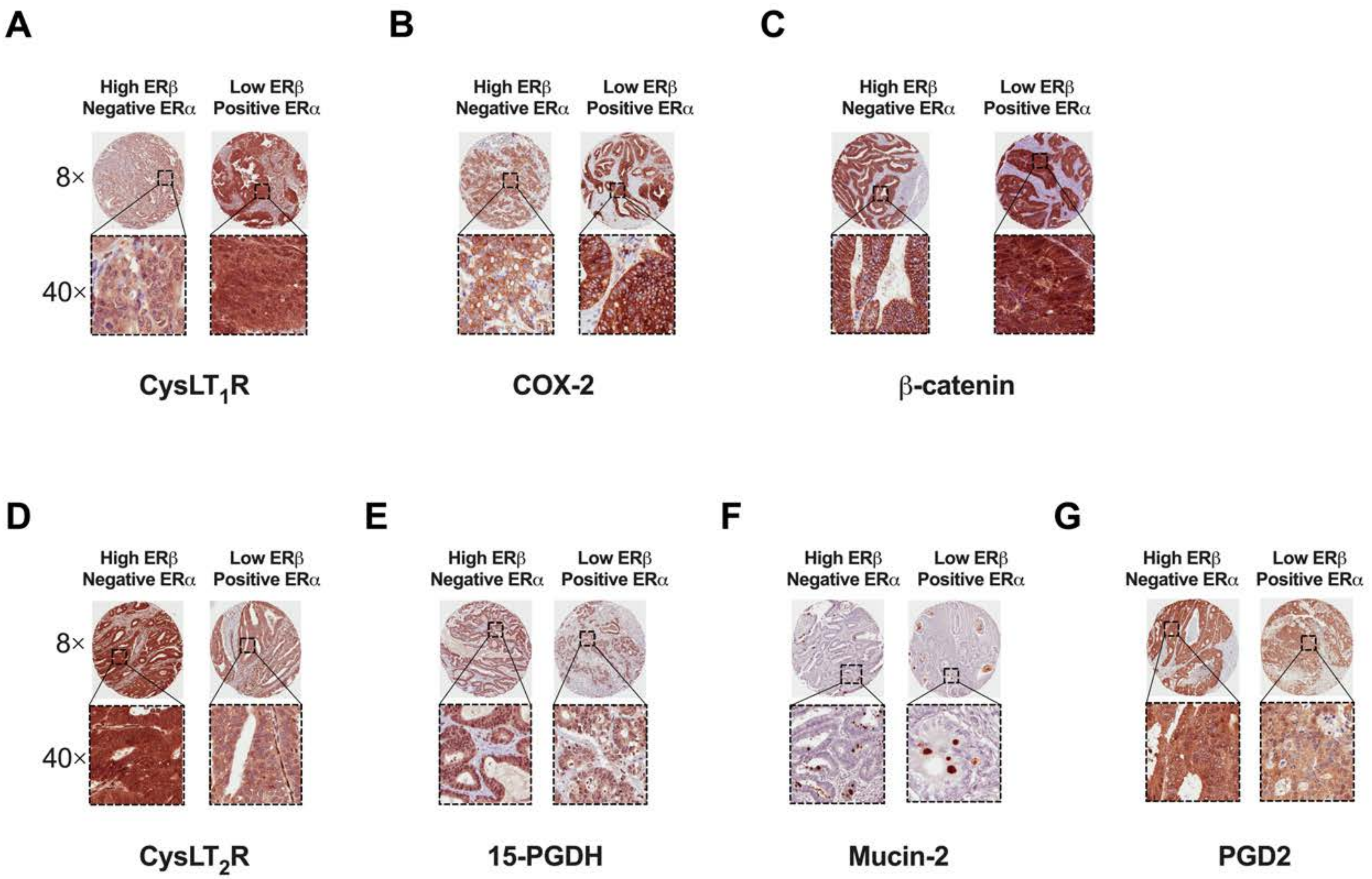

Supplementary figure 3
